# Multi-modal Language models in bioacoustics with zero-shot transfer: a case study

**DOI:** 10.1038/s41598-025-89153-3

**Published:** 2025-02-28

**Authors:** Zhongqi Miao, Benjamin Elizalde, Soham Deshmukh, Justin Kitzes, Huaming Wang, Rahul Dodhia, Juan Lavista Ferres

**Affiliations:** 1 AI for Good Lab, 1 Microsoft Way, Microsoft, Redmond, WA 98052 USA; 2 Microsoft, 1 Microsoft Way, Redmond, WA 98052 USA; 3 Department of Biological Science, University of Pittsburgh, 4200 Fifth Ave, Pittsburgh, PA 15260 USA

**Keywords:** Bioacoustics, Multi-modal Language models, Zero-shot transfer, Audio-Language models, Artificial Intelligence, Wildlife conservation, Biodiversity, Conservation biology, Computer science

## Abstract

Automatically detecting sound events with Artificial Intelligence (AI) has become increas- ingly popular in the field of bioacoustics, ecoacoustics, and soundscape ecology, particularly for wildlife monitoring and conservation. Conventional methods predominantly employ supervised learning techniques that depend on substantial amounts of manually annotated bioacoustic data. However, manual annotation in bioacoustics is tremendously resource- intensive in terms of both human labor and financial resources, and it requires considerable domain expertise. Moreover, the supervised learning framework limits the application scope to predefined categories within a closed setting. The recent advent of Multi-Modal Language Models has markedly enhanced the versatility and possibilities within the realm of AI appli- cations, as this technique addresses many of the challenges that inhibit the deployment of AI in real-world applications. In this paper, we explore the potential of Multi-Modal Language Models in the context of bioacoustics through a case study. We aim to showcase the potential and limitations of Multi-Modal Language Models in bioacoustic applications. In our case study, we applied an Audio-Language Model–—a type of Multi-Modal Language Model that aligns language with audio / sound recording data—–named CLAP (Contrastive Language–Audio Pretraining) to eight bioacoustic benchmarks covering a wide variety of sounds previously unfamiliar to the model. We demonstrate that CLAP, after simple prompt engineering, can effectively recognize group-level categories such as birds, frogs, and whales across the benchmarks without the need for specific model fine-tuning or additional training, achieving a zero-shot transfer recognition performance comparable to supervised learning baselines. Moreover, we show that CLAP has the potential to perform tasks previously unattainable with supervised bioacoustic approaches, such as estimating relative distances and discovering unknown animal species. On the other hand, we also identify limitations of CLAP, such as the model’s inability to recognize fine-grained species-level categories and the reliance on manually engineered text prompts in real-world applications.

**Supplementary Information:**

The online version contains supplementary material available at 10.1038/s41598-025-89153-3.

## Introduction

The world is currently experiencing a rapid loss of global biodiversity due to habitat destruction, climate change, and various human impacts^1–4^. To effectively study and monitor the intricate patterns, underlying drivers, and extensive consequences of these changes, ecologists are increasingly resorting to automated data collection and monitoring methods. By utilizing sensors such as cameras and acoustic recorders, they can now monitor species of interest across spatial and temporal scales previously unattainable^5–11^. 

Among these methods, automated sound recorders (ASRs) or autonomous recording units (ARUs) are being increasingly utilized for surveys of sound-producing animals not easily monitored through image-based devices. This includes animals like birds, frogs, bats, insects, and marine mammals^12–14^. The applications of ARUs are expanding in scope, with some projects now amassing and analyzing hundreds of thousands of audio/ sound recordings^15,16^. Given that manual review of such a vast collection of audio data (i.e., sound recording data) is impractical, automated analysis techniques are essential for extracting valuable ecological information.

Modern artificial intelligence (AI) techniques are increasingly applied to automated detection and localization of bioacoustic events. These techniques involve identifying sound events of interest within audio recordings and providing timestamps for the start and end times of these events^17–24^. In bioacoustics, common approaches for sound event detection and categorical classification often rely on computer vision and deep learning methods like Convolutional Neural Networks (CNNs)^25,26^or Vision Transformers (ViTs)^27,28^within supervised learning frameworks. However, supervised learning depends on a large amount of manually annotated and time-stamped data for every sound of interest. This is necessary to train models capable of producing reliable predictions^23,24,29–31^. Assembling sufficient manually annotated audio data presents challenges mainly due to: (1) the ambiguity in defining the precise beginning and end of bioacoustic events, (2) the need for specialized domain expertise in bioacoustics for accurate annotations, and (3) the typically extended duration of bioacoustic recordings. Consequently, citizen scientists and crowdsourcing labeling services like Amazon Mechanical Turk have been less involved in the annotation of bioacoustic recordings than in labeling datasets from imagery sources, such as those collected by camera traps and satellites^11,32–36^. Moreover, unlike tasks like human speech recognition, field recordings can encompass a broad array of sounds from diverse animals such as birds, frogs, whales, insects, and bats, each requiring specifically annotated datasets for training^37,38^. These constraints not only limit the applicability of supervised models in bioacoustics but also hamper their ability to recognize classes absent from the training set, known as open set or novel species recognition.

The recent emergence of Multi-Modal Language Models^39–44^has spurred a transformative paradigm shift within the realm of AI applications, offering unparalleled model flexibility and potential. These Multi-Modal Language Models primarily focus on aligning language concepts with other forms of data modalities, especially perceptual ones such as images and audio. This alignment marks a stark difference from traditional machine learning models that rely on supervised learning, which emphasizes sample-to-label mapping^45^, or unsupervised learning, which lacks a direct connection to language semantics^46,47^. This paradigm shift opens up new possibilities for innovative solutions to overcome the challenges posed by current supervised-learning techniques in bioacoustics sound event detection: the dependency on extensive manually annotated data and the limitation to a predefined label space in a restricted setting.

One of the most noteworthy advancements of the Multi-Modal Language Model technique is its zero-shot recognition capability (i.e., being able to recognize categories without seeing similar data during training)^39,48,49^. This paper explores this zero-shot capability of Multi-Modal Language Models in the context of bioacoustics through a case study that employs an Audio-Language Model–a type of Multi-Modal Language Model that aligns language with audio data–named CLAP (i.e., Contrastive Language-Audio Pretraining)^50^. We’ve applied CLAP to eight different bioacoustics benchmarks of group-level categories such as birds, frogs, whales, meerkats, and gun-shots, curated from established bioacoustics datasets such as BEANS^51^, Warblr^18^, and Freefield^52^. Our experiments show that, after simple prompt engineering, CLAP exhibits comparable recognition performance to fully supervised baselines on six out of the eight bioacoustics benchmarks without dedicated model fine-tuning, despite these benchmarks being novel to the model. Additionally, CLAP shows promise in tasks such as recognizing unknown or novel animal species and estimating relative distances, all without the requirement of dedicated model training. On the other hand, we also identify limitations of CLAP and Multi-Modal Language Models in general in the applications of bioacoustics, such as the model’s inability to recognize fine-grained species-level categories and the challenges of using manually engineered text prompts in real-world applications. We then propose potential future research directions to address these limitations. The objective of this paper is to introduce the Multi-Modal Language Model technique to the bioacoustics community and to examine its potential and limitations.

## Methods

### Multi-modal Language models

Recent years have witnessed a surge of interest in the study of multi-modal models, primarily because of their unique ability to process and generate a range of data modalities simultaneously–including vision, audio, and language^39,50^. Among the various combinations of multi—modalities^53–55^, Multi-Modal Language Models^39–43^have become especially prominent. These models mainly focus on aligning language concepts with other data modalities (e.g., imagery and audio)^39,50^, an area of research invigorated by the successes of large language model (LLMs) development^44^.

**Fig. 1:**
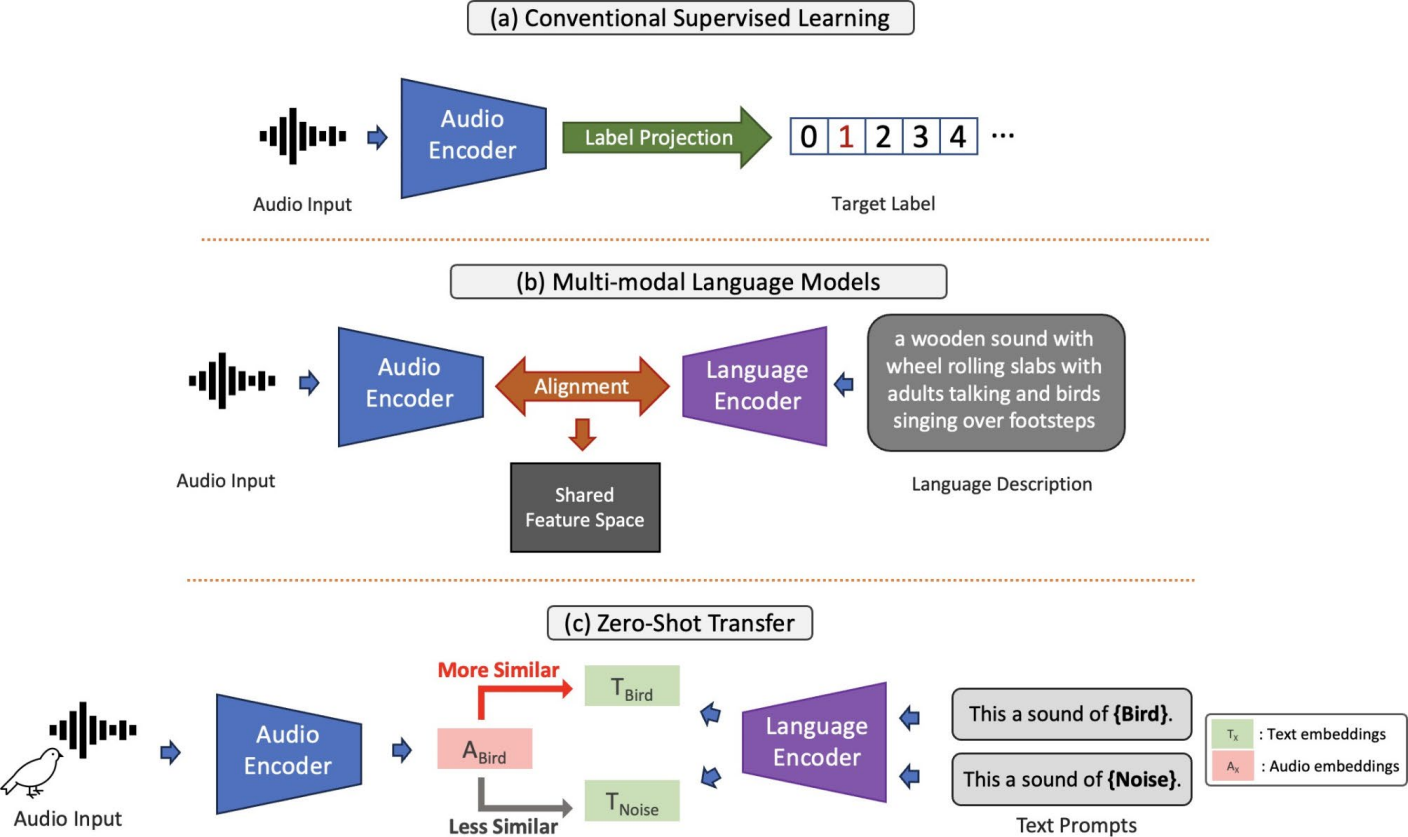
(**a**) In conventional supervised learning, input samples are typically mapped to digital labels, often discrete in nature for categorical classification. Each label represents a single category, and there are no inherent semantic relationships encoded within this labeling system. Furthermore, all categories must be explicitly defined prior to training and remain unchanged during inference, leading to considerable limitations on the applications of such models in real-world. (**b**) Multi-Modal Language Models (Audio-Language Models in this example) align audio embeddings and their corresponding language description embeddings into a shared feature space. This learning paradigm does not rely on fixed sets of predefined categories as text descriptions are usually unique to each audio sample and are not confined to categorical concepts. In the above text description example, not only are concepts of “*wheel rolling*”, “*adults talking*”, and “*birds singing*” encoded, but relational concepts like “*over footsteps*” are also encoded and associated with corresponding sounds. (**c**) In the absence of categorical labels in training and due to the similarity-based nature of this learning paradigm, we can define a set of text categories during inference (*Bird* and *Noise* in this example) to determine which language embedding of these post-defined categories the audio sample is most similar to. In the above example, the embedding of a bird audio is more similar to the language embedding of the text prompt, “*This is a sound of Bird.*” Consequently, we can classify this audio as a sound of birds.

Traditional supervised learning protocols often struggle with mapping training data to language concepts or semantics^39,41^. For instance, categorical supervised learning labels data with discrete numerical labels, which often oversimplifies the complex language concepts they aim to represent. Take ImageNet^56^, one of the most widely applied datasets for image classification, as an example; even though there are 120 categories of dog breeds in the dataset, these categories are indistinguishable from categories such as *cars* or *fish* when encoded as discrete labels (Fig. 1(a)). Ultimately, this oversimplification leads to artificial decision boundaries within a continuous feature space, complicates the learning of semantic relationships, and often confines the models to predefined label spaces^45^.

In contrast, Multi-Modal Language Models circumvent these limitations by directly aligning features of data modalities with language concepts through maximizing the feature similarities of different data modalities^39^. This results in a continuous feature space that inherently encodes semantic relationships, thanks to the natural semantic continuity facilitated by the learning process in LLMs. For instance, in Vision-Language Models (a type of Multi-Modal Language Model that aligns vision and language features), the visual features of both the *Golden Retriever* and *Border Collie* breeds are compelled to exhibit closer proximity in the feature space, even before the models generalize from visual similarities, due to the presence of language features. This process enables the model to group data from both categories as subspecies of *dogs*, highlighting their semantic difference from categories like *cars*. Additionally, the continuous semantic feature space facilitates nuanced recognition and data generation tasks^41,42^. For example, it becomes feasible to identify unique amalgamated concepts, such as *dog-like cars*, without requiring dedicated training data, as long as the concepts of *dogs* and *cars* are sufficiently represented and appropriately aligned with the language features in the multi-modal feature space. Consequently, Multi-Modal Language Models are free from traditional sample-to-label mapping because categorical labels for project-specific inference can be defined after the models are trained (see Fig. 1(b) and (c)).

In the context of bioacoustics applications, during inference, various sets of categories can be defined in words or descriptive phrases to suit the requirements and interests of a specific project after an Audio-Language Model (a Multi-Modal Language Model that aligns audio and language features) is trained (e.g., *bird songs* and *noise*). Categorical classification is conducted by measuring the similarities between the feature embeddings of the testing samples and the text embeddings of these post-defined words and phrases and does not rely on predefined decision boundaries^39^. Particularly, if the training data contain bird calls, regardless of the species, an Audio-Language Model has the potential to recognize calls from almost any bird species that produce regular-sounding calls, including those not present in the training data, by setting up a post-defined category called *bird* during inference. The Audio-Language Model compares the feature/embedding similarity of the testing audios (i.e., sound recordings) with existing semantic concepts learned through LLMs and summarize the samples into categories (*bird* in this case). Since the model does not have a specified *bird* category during training, and this post-defined category can be applied to any unseen datasets with bird calls, this process is known as *Zero-Shot Transfer*^39^ (Fig. 1(c)). The ability to define categories post-training offers substantially greater flexibility compared to traditional supervised learning approaches, which restrict models to predefined labels^45^, and unsupervised or self-supervised learning, where models are unable to generate feature spaces linked to semantic or text categories^46,47^.

Moreover, post-defined categories (or text prompts, to be more specific^39^) are not limited to single categorical definitions. Text prompts can be any combinations of phrases or existing semantics in the LLMs. For instance, they might include *“This is the sound of an animal mumbling”* or *“This is the sound of a bird singing in the background.”* Such descriptive text prompts allow researchers to undertake certain tasks that are not easily achievable with traditional AI methods in bioacoustics, such as relative distance estimation and the discovery of animal species (i.e., detecting sound events of semantics that do not exist in pre-trained models for novel categories).

In this paper, we explore the zero-shot transfer and recognition abilities of Multi-Modal Language Models in bioacoustics through a case study. The details of the experiments are in the following sections.

### Contrastive Language-Audio Pretraining (CLAP)

In this project, we use CLAP (Contrastive Language-Audio Pretraining)^50^as our multi-modal model for the case study on eight bioacoustics datasets curated from established bioacoustics benchmarks such as BEANS^51^, Warblr^18^, and Freefield^52^. These datasets are manually annotated and validated by human experts in their original studies. CLAP is an Audio-Language Model (ALM)^39,50,57,58^ that aims to align (i.e., enhance the embedding similarities) features of audio samples and their associated text descriptions through learning from large quantities of audio-text pairs. These audio-text pairings are unique to each pair of samples and are not restricted to specific categories. Therefore, any relevant text description and audio clip can be used to train the model, allowing for the inclusion of extensive online data sources in the training process.

A trained CLAP model can identify audio samples that are similar to those it has encountered during training and associate them with semantic concepts learned from the corresponding training text descriptions. This means that the model can use any relevant words semantically similar to the existing training text descriptions and associate these words (i.e., post-defined categories) with the test audios, enabling the procedure of zero-shot transfer. The whole process is solely similarity-based, therefore, no decision boundaries are learned during training, and the inference is conducted by measuring the similarity between the test audio embeddings and the text embeddings of the post-defined categories (Fig. 1 (c)).

Technical details of CLAP and Zero-Shot Transfer are provided in the Appendix.

### Datasets for CLAP pretraining

CLAP is trained using audio-text pairs, rather than traditional audio-label datasets. These pairs are sourced from various standard audio datasets that span different domains, including environmental sounds, speech, emotions, actions, and music. Even though these datasets were not explicitly annotated for bioacoustics research, they still encompass a wide range of animal sounds such as those of lions, tigers, birds, dogs, wolves, rodents, insects, frogs, snakes, and whales. This diversity of sound sources enables CLAP to effectively perform Zero-Shot Transfer on most bioacoustics benchmarks. However, since most of the standard data do not have detailed animal species-level annotations, and it is challenging to collect audios for every single possible animal species on Earth, CLAP does not have the ability to differentiate species-level sounds, out-of-the-box. Instead, it is possible to recognize group-level sounds, such as *birds*,* whales*,* frogs*, and *meerkats*.

In this project, we train CLAP with a Transformer-based audio encoder (***Hierarchical Token Semantic Audio Transformer***,*** HTS-AT***^59^) with different numbers of data for performance comparisons on the scales of pretraining data. We also have a third version of CLAP pretrained with a CNN-based audio encoder (***Pretrained Audio Neural Networks***,*** PANN***^60^) and a smaller scale of pretrained data for a fully zero-shot experiment (i.e., even similar sounding calls from similar animals do not exist in the training data), and faster experiment turnover rate.

The details of the pretraining audio-text datasets for the three versions of CLAP are listed below:

- CLAP-HTS-AT (450 K): For the HTS-AT-based model, the first version is pretrained on 450,000 audio- text pairs curated from FSD50k^61^, ClothoV2^62^, AudioCaps^63^, and MACS^64^, SoundDescs^65^, BigSoundBank^66^, SoundBible^66^, FMA^67^, NSynth^68^, and findsound.com.

- CLAP-HTS-AT (2.1 M): The second version of HTS-AT-based CLAP is pretrained with additional 1,650,000 audio-text pairs (2.1 million audio-text pairs in total) curated from CMUMOSI^69^, MELD^70^, IEMOCAP^71^, MOSEI^69^, MSPPodcast^72^, CochlScene^73^, AudioSet (Filtered)^74^, Kinetics700^75^, Freesound^76^, and ProSoundEffects^77^.
- CLAP-PANN (128K): The smaller scaled PANN-based model is pretrained on 128,000 audio-text pairs curated from FSD50k^61^, ClothoV2^62^, AudioCaps^63^, and MACS^64^ for faster experiment turnover.

Additional information about these datasets can be found in the Appendix.

### Supervised baselines

Since existing supervised bioacoustics benchmarks such as The Benchmarks of Animal Sounds (BEANS)^51^are mostly about species level recognition, we need to prepare group-level supervised benchmarks for the evaluation of CLAP. We use ResNet-18^78^as our supervised learning baseline model, primarily due to the relatively small dataset sizes present in most of the benchmarks within this project, as well as its broad application in both bioacoustic research and other AI conservation projects^51^. We didn’t choose larger deep learning models because it has been reported that deeper models, such as ResNet-50^78^, Inception^79^, and Vision Transformers^27,28^, tend to overfit more easily on smaller datasets and their performance gains are often limited^80^. Nevertheless, we demonstrate that ResNet-18 offers a representative example of fully supervised performance across all benchmarks.

### Benchmark datasets

We use seven public bioacoustics benchmark datasets to demonstrate the out-of-the-box group-level zero-shot transfer animal sound recognition ability of CLAP across different sound sources. There are four datasets for bird call detection (Jackdaw^81^, Enabirds^82^, Freefield^52^, and Warblr^18^), one dataset for both birds and frogs (Rfcx^83^), one for Minke whales (Hiceas^51^), and one for meerkats (Meerkat^81^).

We also include a benchmark dataset for gunshot detection in the rain forest area (Tropical-Gunshots^84^). Although the focus of this dataset is not exclusively on bioacoustics, the detection of gunshots in the wild—particularly in rain forests–shows promise as an automated approach for anti-poaching efforts. Incorporating this dataset al.lows us not only to broaden the applications of CLAP within the realm of general animal audios but also to assess its capability to generalize across a spectrum of sounds, including those not related to animals.

None of these benchmarks have been used in CLAP’s training, and there are distinct quality and perceptual differences, or domain discrepancies, between these benchmarks and CLAP’s training datasets. In addition, there is no guarantee that there is an overlap on the animals species between these benchmarks and CLAP’s training data. In other words, these benchmarks are considered unfamiliar and novel to CLAP. Furthermore, sounds of meerkat, or any other relevant species, do not exist in the 128k training audio-text pairs for the CLAP-PANN model making meerkat sounds completely unknown to the smaller scaled model. Therefore, we use the PANN model on the Meerkats data for a fully zero-shot experiment.

In our experiments, we utilize the training-validation splits defined by the original studies of these datasets^18,51,52,84^ to train and validate the baseline supervised models. The testing splits, also defined by each original study, are used to evaluate the performance of both CLAP and the supervised baselines.

For the Jackdaw, Enabirds, Rfcx, Hiceas, and Meerkats datasets, since they are directly from the BEANS benchmark, we segment the audios following BEANS’ default definition of window sizes. The Freefield, Warblr, and Tropical-Gunshots datasets are already pre-segmented, therefore, we directly use the audio segments in our experiments. We resample all the benchmarks to 44.1 kHz using the PyTorch default Sinc interpolation method to match the CLAP models’ training data and avoid statistical discrepancies between the training and testing data. Details of all the datasets can be found in the Appendix.

Since most of these benchmarks are originally species-level, we group them into group-level categories such as *birds*, *frogs*, *whales*, and *meerkats* to evaluate CLAP’s performance. We label each segment from the majority of benchmarks as either positive (indicating the presence of events of interest) or negative (indicating the absence of such events). However, the Rfcx dataset, which comprises recordings of both bird and frog sounds, is treated differently. We utilize the Rfcx dataset to assess CLAP’s capability to differentiate between distinct animal species (birds or frogs in this case). Consequently, we conduct experiments exclusively using sound segments containing bird and frog sounds. Within this benchmark, a segment is annotated as positive if it contains only bird sounds, and as negative if it includes only frog sounds. In essence, the Rfcx experiments aim to demonstrate CLAP’s recognition ability in a dataset that features multiple groups of animal sounds.

### Experiment settings

**Fig. 2:**
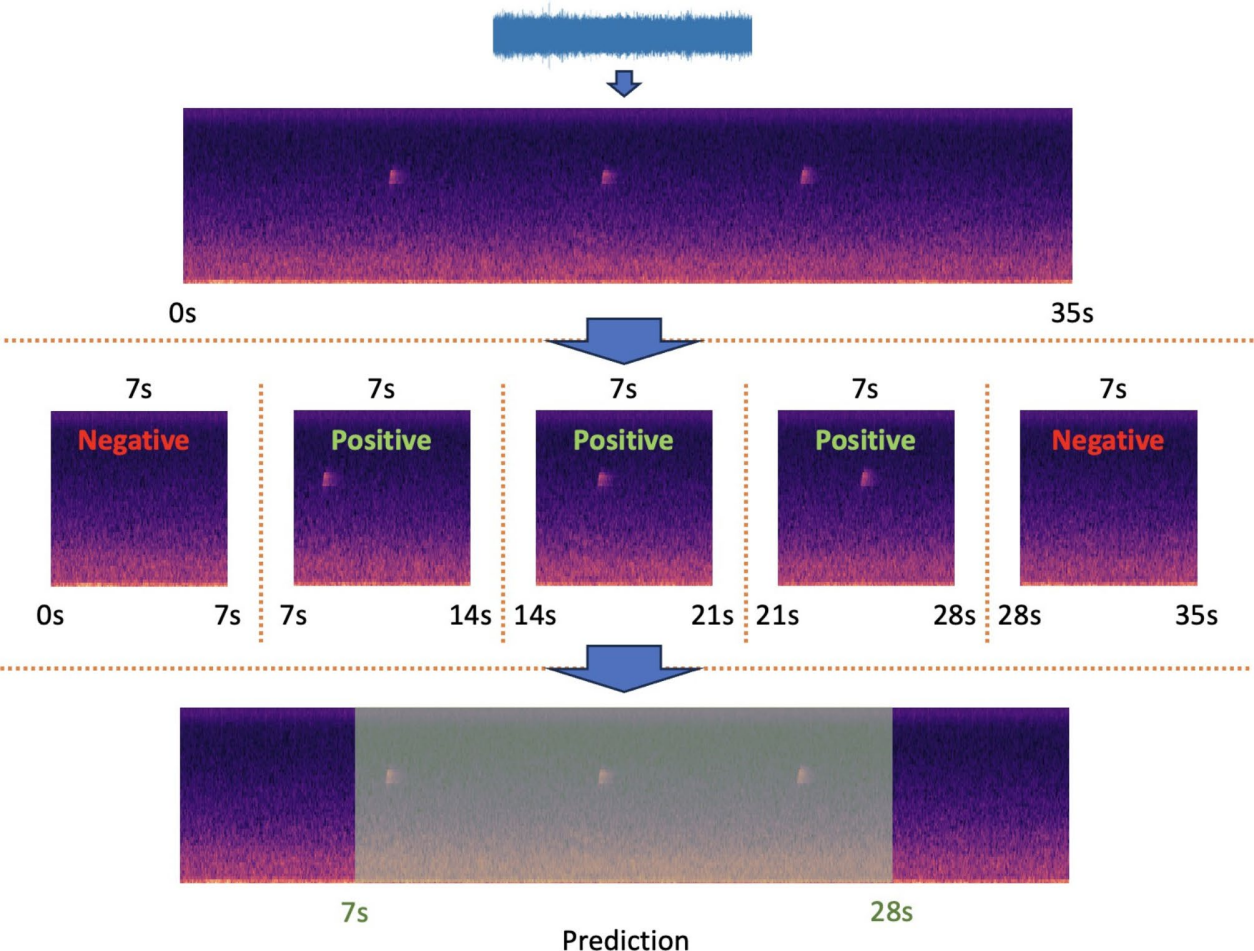
Illustration of fixed window sound event existence classification. In bioacoustics, a common approach to detect sound events of interest is classification of audio segments with fixed window sizes. The usual procedure begins with the conversion of raw audio into a visual representation, such as a spectrogram. Subsequently, the spectrogram is divided into segments using a fixed time window (e.g., 7 s in this example) and a window step size (e.g., also 7 s in this example). By employing a visual classification model, the presence or absence of the sound event of interest is predicted for each segment. Using these predictions, we can obtain approximate time stamps for the localization of sound events. In practice, step sizes are often smaller than window sizes for higher classification resolution. For example, under the BEANS setup, the Jackdaw benchmark has a 2-second window size with a 1-second step size^51^.

Following BEANS, we simplify the timestamp-based sound event detection task by converting it into a sound event existence classification task within fixed-window-size audio segments of long recordings–a common approach in bioacoustic research (see Fig. 2^18,20,21,51^). This method circumvents the technical challenges associated with predicting the exact start and end times of sound events in audio recordings. Specifically, we use the CLAP model as a classifier for sound events and evaluate its performance on eight bioacoustic benchmark datasets that are new to CLAP.

We employ the Zero-Shot Transfer protocol^39^ to evaluate CLAP’s performance (see Fig. 1(c)). Specifically, during the inference or testing phase, we utilize data the model has not encountered during training and define categories using text prompts post-training under the setting of zero-shot transfer. To demonstrate CLAP’s capability at detecting post-defined sound event categories, we compare its performance against fully supervised baseline models that have been fine-tuned on benchmark datasets, which serve as the upper bounds.

For evaluation, we use Average Precision (AP)^51^. The details of how we calculated these metrics are reported in the Appendix.

### Text prompts for zero-shot transfer

In order to perform zero-shot transfer, Audio-Language Models like CLAP require text prompts to define the categories for recognition post training. In addition, instead of fully automated classification, manually engineered text prompts for each benchmark are also needed to ensure optimal performance. We identify this as one of the biggest challenges in deploying Multi-Modal Language Models in real-world applications^85,86^.

However, in this project, our main objective is to demonstrate the potential of Multi-Modal Language Models in bioacoustics, therefore, we manually engineer text prompts for each benchmark using the validation sets. The text prompts for each benchmark are detailed in Table 1.

## Results

In this section, we present the results of benchmark comparisons between CLAP and supervised baselines, along with corresponding discussions on the experimental details of CLAP, limitations of the technique, and future directions.

### Zero-shot transfer

Table 2 shows that the two CLAP models exhibit overall comparable AP to the fully supervised ResNet-18 baselines on most benchmarks, without the need for model fine-tuning or additional training on the target. datasets. Furthermore, the Transformer-based CLAP model pre-trained with 2.1 million audio-text pairs (CLAP-HTS-AT 2.1 M) performs better than or equally to the supervised baselines on six out of the eight benchmarks. These results underscore the potential of CLAP to detect bioacoustic signals from a variety of sound sources after simple prompt engineering, provided that the model has been exposed to relevant concepts such as birds, whales, frogs, and gunshots during pre-training, regardless of the species.

In addition, the comparison between the two HTS-AT models shows that the 2.1 M model consistently outperforms the 450 K model, with an average AP increase of 0.05. This suggests that utilizing more pretraining data can enhance the performance of such foundational multi-modal models.

**Table 1:** Text prompts to perform zero-shot classification for each benchmark.

#	Benchmarks	Text prompts
1	Jackdaw	Is this a sound of birds chirping or noise?
2	Freefield	Is this a sound of birds chirping or noise?
3	Warblr	Is this a sound of birds chirping or noise?
4	Rfcx	Is this a sound of birds singing far in the background or frogs?
5	Hiceas	Is this a sound of whale vocalizations or noise?
6	Enabirds	Is this a sound of birds chirping or noise?
7	Meerkat	Is this a sound of meerkats clucking or non-animal noise?
8	Tropical-Gunshots	Is this a sound of gunshots in the distance or broken branches and noise?

**Table 2:** Average Precision (AP) comparisons between CLAP (growing number of pretraining pairs) and supervised baselines. Higher is better.

Settings	Models	Jackdaw	Freefield	Warblr	Rfcx-Bird	Rfcx-Frog	Hiceas	Enabirds	Meerkat	Tropical-Gunshots
Supervised	ResNet-18	0.99	0.83	0.96	0.88	0.79	0.30	0.98	0.94	0.64
Zero-Shot Transfer	CLAP-HTS-AT (450 K)	0.95(↓)	0.82(↓)	**0.96(-)**	0.70(↓)	0.78(↓)	0.29(↓)	0.96(↓)	0.81(↓)	0.49(↓)
	CLAP-HTS-AT (2.1 M)	0.96(↓)	**0.84(**↑**)**	**0.96(-)**	0.79(↓)	**0.81(**↑**)**	**0.30(-)**	**0.98(-)**	0.87(↓)	**0.67(**↑**)**

#### Source window size matters in audio segment classification

Transformer models typically rely on consistent input audio length between training and testing for optimal performance. The default input window size for our HTS-AT model during pretraining is seven seconds. Consequently, to utilize HTS-AT effectively, we must duplicate each input audio segment if the source samples are shorter than seven seconds, or truncate the audio if the samples exceed seven seconds. This process can result in unnatural sound duplication or lost information, potentially leading to degraded performance.

Table 3 presents performance comparisons across different source audio window sizes (i.e., the original audio segment length of the data). The HTS-AT models shows improvements when using a seven-second source audio window, compared to using either truncated or duplicated samples. Notably, on the Meerkat benchmark, the performance of the 2.1 M model increases from an AP of 0.87 to 0.98. Longer source window sizes remove unnatural audio duplications, and Transformer-based CLAP models perform better with these settings, achieving performance comparable to that of supervised baselines.

**Table 3:** Average Precision (AP) comparisons of different source window sizes on BirdVox and Meerkat. Higher is better.

Settings	Models	Rfcx-Bird 10-sec window 7-sec window		Meerkat 2-sec window 7-sec window	
Supervised	ResNet-18	0.88	0.89	0.94	0.97
Zero-Shot Transfer	CLAP-HTS-AT (450 K)	0.70(↓)	0.72(↓)	0.81(↓)	**0.97(-)**
	CLAP-HTS-AT (2.1 M)	0.79(↓)	0.82(↓)	0.87(↓)	**0.98(**↑**)**

### The importance of text prompts

One of the crucial factors affecting the performance of Multi-Modal Language Models, such as CLAP, is the engineering of text prompts. Changing text prompts can result in vastly different model prediction performance and can even enable the model to perform tasks beyond categorical classification.

#### Detailed descriptions can improve model performance

Table 1 shows that Rfcx-Birds and Tropical-Gunshots have relatively more complex text prompts compared to other benchmarks. Specifically, we use *“birds singing far in the background”* as the text prompt for CLAP to recognize most of the bird calls in the Rfcx-Birds dataset. As presented in Table 4a, using *“birds”* alone as the text prompt results in poor performance (0.54 AP) due to the dataset’s noisy nature. However, adding descriptive words such as *“singing”* improves recognition performance (0.63 AP). The most substantial improvement in performance is observed when we include the concept *“in the background”* (0.73 AP). And the word *“far”*further improved the performance (0.79 AP). Furthermore, the effectiveness of these text prompts suggests that CLAP is capable of differentiating between foreground and background sounds without specialized training in relative distance estimation, which represents a significant challenge in bioacoustics owing to limited training data^87^. However, there are still limitations to this capability, as the model may not be able to provide precise numerical distances of sound sources due to the similarity based mechanism of Multi-Modal Language Models. Therefore, there is still a long way before practical applications.

Similar patterns can be observed in the results for the Tropical-Gunshots dataset (Table 4b). Detecting gunshots within rain forests poses a significant challenge due to an array of similar-sounding events. For example, our analysis reveals that the most common sources of confusion, closely resembling gunshot sounds, are those of breaking tree branches. To mitigate this issue, we introduce the term *“broken branches”* to refine the characterization of non-gunshot sounds in the dataset. This enhancement leads to an improved zero-shot transfer performance, achieving a 0.67 AP, which surpasses the 0.64 AP of the supervised baseline.

#### Novel categories and species discovery

As mentioned in the Methods section, we have a smaller scaled PANN model that is trained on 128,000 audio-text pairs that do not contain any meerkat-related audios for a fully zero-shot experiment. Table 4c shows the performance of the CLAP-PANN (128 K) model on the Meerkat dataset, utilizing various text prompts to detect meerkats. The sounds associated with meerkats being absent in the pretraining data means that the direct use of *“meerkats”* as the prompt key is ineffective for CLAP’s meerkat detection, resulting in a 0.56 AP. Yet, the integration of descriptive terms like *“clucking”* markedly improves the recognition performance to a 0.80 AP, up from 0.56 AP. This rise does not suggest that the model comprehends the concept of *“meerkats*,*”* given that the single use of *“clucking”* yields a superior 0.82 AP, surpassing the result of using the combined prompt *“meerkats clucking*,*”* which is 0.80 AP.

Nevertheless, the CLAP-PANN model does possess the concept of *“animal”*. By combining *“animal”* with descriptive words like *“clucking”* and *“growling*,*”* the model achieves its highest meerkat recognition performance at 0.88 AP in our experiments. These findings suggest that it is possible to narrow down targets and detect the majority of meerkat sounds, even without prior audio knowledge specific to meerkats within the model. In other words, when provided with appropriate descriptive words, CLAP has the potential (through the similarity calculation between features of input audio and language prompts) to identify previously unknown or ambiguous animal species in real-world scenarios.

## Discussion

In this section, we discuss some of the limitations we have identified in the applications of CLAP and Multi-Modal Language Models in general, as well as potential future directions for improvements.

### Prompt-engineering-free zero-shot models

Based on the previous discussions, it is clear that the quality of manually engineered text prompts directly impacts the zero-shot recognition performance of CLAP making such methods far from practical deployment at the moment. In other words, even though the model can recognize unseen samples in a zero-shot manner, the performance is highly dependent on the quality of the text prompts human experts give to the model. However, besides manual prompt engineering on annotated validation datasets, there currently exists a lack of efficient and effective methods to acquire high-quality, detailed text prompts. In addition, the English language contains hundreds of words that can describe sounds, and some of these words may even yield better performance for tasks like meerkat identification. There is no straightforward method for conducting a large-scale vocabulary search either, as the number of possible word combinations could be practically infinite and it largely depend on the user’s own knowledge and vocabulary Consequently, most studies on Multi-Modal Language Models can only offer limited and sometimes anecdotal evidence based on manual prompt engineering^88^. In addition, the reliance on manual prompt engineering also largely restricts the practical application of Multi-Modal Language Models in real-world scenarios under zero-shot settings, as the process can be time-consuming and labor-intensive, especially when dealing with large-scale bioacoustic datasets. Furthermore, since the majority of existing Multi-Modal Language Models are primarily trained on English^44^, this limits the use of such methods in non-English-speaking communities. These limitations underscore the need for future research to develop prompt-engineering-free zero-shot models that can automatically generate high-quality text prompts for zero-shot recognition tasks. Although several attempts at automatic text prompt generation have been made in the Vision-Language model domain, such as with K-LITE^89^and LENS^90^, there remains a notable gap in studies focused on the Audio-Language Model domain. This identifies a promising avenue for future research in this area.

**Table 4:** Experiment results on text prompts.

**(a) CLAP-HTS-AT (2.1M) performance of recognizing birds in the background. Higher is better**			
Is this a sound of *{}* or frogs?		Ap	
Birds Birds singing Birds singing in the background Birds singing ***far*** in the background Supervised baseline ap:		0.54 0.63 0.73 **0.79** 0.88	
**(b)** **CLAP-HTS-AT (2.1 M) performance of recognizing gunshot sounds in tropical rain forest. Higher is better**			
Is this a sound of *{*A*} *or *{*B*}*?		Ap	
A: Gunshots, B: Noise A: Gunshots in the distance, B: Noise A: Gunshots in the distance, B: Broken branches or noise Supervised baseline ap:		0.36 0.57 **0.67** 0.64	
**(c) CLAP-PANN (128 K) performance of recognizing meerkat sounds using 2-second window**			
Is this a sound of *{}* or non-animal noise?	Ap		
Meerkats Meerkats growling Meerkats clucking Meerkats clucking or growling Growling Clucking Clucking or growling Animals Animals growling Animals clucking Animals clucking or growling Supervised baseline ap:	0.56 0.68 0.80 0.79 0.63 0.82 0.78 0.85 0.82 0.86 **0.88** 0.94		

### Dedicated bioacoustics datasets for ALMs

Since the current version of CLAP, as well as most other Audio-Language Models^91^, is trained mostly on urban and standard audio datasets, one approach to enhance performance on bioacoustic tasks is to incorporate bioacoustic datasets directly into the existing pool of training data. This inclusion would also account for ultrasonic sounds from animals like dolphins and bats that are currently unsupported. Such integration could enable the models to exhibit better generalization abilities when applied to diverse bioacoustic tasks. However, training Multi-Modal Language Models relies on the availability of text descriptions associated with the audio files. To the best of our knowledge, datasets that pair bioacoustic audio with descriptive text do not yet exist. Thus, the development of such datasets represents a promising direction for future progress in Multi-Modal Language Models for bioacoustics.

On the other hand, collecting large-scale, real-world bioacoustics datasets with accompanying text de- scriptions can be challenging. An alternative is the creation of large-scale synthetic datasets, which are also currently nonexistent. Despite the discrepancy between synthetic and real-world data, exploring the possibility of using synthetic data to train ALMs is still promising, considering the accessibility and control that synthetic datasets can provide.

### Species-level recognition

Even though group-level recognition can work as a noise/empty filtering tool helping practitioners to focus on the most relevant parts of the audio recordings, it is still essential to recognize species-level categories for more detailed ecological studies. The current iterations of CLAP and other Audio-Language Models are not yet capable of recognizing fine-grained species-level categories, such as distinguishing between different species of woodpeckers or thrushes. The biggest reason is because the current training data set does not have any fine-grained species names in its language descriptions. Therefore, there is no way the model can link species names with audio features, just like the word “meerkat”. Even within the Vision-Language model domain, fine-grained zero-shot recognition remains one of the biggest challenges and only preliminary studies have been carried out thus far^90,92^. Yet, the advancements in Vision-Language Models on fine-grained zero-shot recognition suggest that this task is possible with high quality of text prompts. For instance, the Kosmos-1^41^ project has shown that detailed verbal descriptions can aid in distinguishing between similar animal species in images, like those of a three-toed woodpecker versus a downy woodpecker. How to transfer such techniques into the bioacoustics domain is a potential direction for future research on species-level zero-shot recognition with Audio-Language Models.

## Supplementary Information

Below is the link to the electronic supplementary material.

Supplementary material 1 (DOCX 152.7 kb)

## Data Availability

All the datasets used in this project are published datasets. Upon the publication of this manuscript, a merged dataset combining all the datasets mentioned in the paper will be made available.

## Code availability

Code for this project is ready for peer review through Zenodo: https://zenodo.org/records/10565262 and will be published upon acceptance through Github: https://github.com/zhmiao/ZeroShotTransferBioacousticsCode.
