# Supplementary material for: Multi-modal Language models in bioacoustics with zero-shot transfer: a case study
Source: Sci Rep. 2025 Feb 28;15:7242. doi: 10.1038/s41598-025-89153-3 (PMC11871236; doi:10.1038/s41598-025-89153-3)
Supplement: Supplementary file 1 — Supplementary material 1 (DOCX 152.7 kb) [file 41598_2025_89153_MOESM1_ESM.docx]

Appendix for Multi-Modal Language Models in Bioacoustics with Zero-shot Transfer: A Case Study

Zhongqi Miao^a^, Benjamin Elizalde^b^, Soham Deshmukh^b^, Justin Kitzes^c^, Huaming Wang^b^, Rahul Dodhia^a^, and Juan Lavista Ferres^a^

^a^AI for Good Lab, Microsoft, 1 Microsoft Way, Redmond, WA, USA, 98052 ^b^Microsoft, 1 Microsoft Way, Redmond, WA, USA, 98052 ^c^Department of Biological Science, University of Pittsburgh, 4200 Fifth Ave, Pittsburgh, PA, USA, 15260

December 16, 2024

# Appendix

## Technical background

In this section, we provide technical backgrounds for CLAP.

### Contrastive Language-Audio Pretraining (CLAP)

Multi-Modal Language models are not conventional self-supervised learning techniques as their learning is not based solely on the samples themselves but also through a similarity mapping between perception and text features. This also makes them different from traditional supervised learning methods that use sample-to-label mapping, since text descriptions are often unique to each sample. Thanks to the existence of language feature embeddings, language-based multi-modal models are not limited to pretraining but can be applied directly as foundation models [1] to downstream tasks such as classification.

CLAP is an ALM model that maximizes the similarities between feature embeddings extracted from raw samples (images or audios) and features extracted from the corresponding text descriptions through LLMs (Supplementary Fig. 1). Let the processed audio be$X_{a}$ s.t. $\begin{aligned} X_{a}\in\mathbb{R}^{F\times T} \end{aligned}$ where *F* are the number of spectral components (e.g. Mel bins) and *T* are the number of time bins. Let the text be represented by $\begin{aligned} X_{t} \end{aligned}$. Each audio-text pair in a batch of *N* is represented as $\begin{aligned} \{X_{a},X_{t}{\}}_{i} \end{aligned}$where $\begin{aligned} i\in[1,N] \end{aligned}$. For convenience, we drop the *i* notation, and henceforth $\{X_{a},X_{t}\}$ will denote a batch of N.

From the pairs, the audio and text are passed through an audio encoder and a text encoder respectively. Let $\begin{aligned} f_{a}(.) \end{aligned}$ represent the audio encoder and $\begin{aligned} f_{t}(.) \end{aligned}$ represent the text encoder. For a batch of N:

$\begin{aligned} {\overset{^}{X}}_{a}=f_{a}(X_{a});{\overset{^}{X}}_{t}=f_{t}(X_{t}) \end{aligned}$ (1)

where$\begin{aligned} {\overset{^}{X}}_{a}\in\mathbb{R}^{N\times V} \end{aligned}$ are the audio representations of dimensionality *V*, and $\begin{aligned} {\overset{^}{X}}_{t}\in\mathbb{R}^{N\times V} \end{aligned}$ are the text representations of dimensionality *U*.

We brought audio and text representations, $\begin{aligned} {\overset{^}{X}}_{a} \end{aligned}$ and $\begin{aligned} {\overset{^}{X}}_{t} \end{aligned}$, into a joint multi-modal space of dimension *d* by using a learnable linear projection:

$\begin{aligned} E_{a}=L_{a}(X_{a});E_{t}=L_{t}(X_{t}) \end{aligned}$ (2)

where $\begin{aligned} E_{a}\in\mathbb{R}^{N\times d} \end{aligned}$, $\begin{aligned} E_{t}\in\mathbb{R}^{N\times d} \end{aligned}$, $L_{a}$ and $L_{t}$ are the linear projections for audio and text respectively.

Now that the audio and text embeddings ($E_{a}$, $E_{t}$) are comparable, we can measure similarity:

$\begin{aligned} C=\tau(E_{t}\cdot E_{a}^{\top}) \end{aligned}$ (3)

where τ is a temperature parameter to scale the range of logits. The similarity matrix $\begin{aligned} C\in\mathbb{R}^{N\times N} \end{aligned}$ has *N* correct pairs in the diagonal and $\begin{aligned} N^{2}-N \end{aligned}$ incorrect pairs in the off-diagonal.

$\begin{aligned} \mathcal{L}=0.5(\mathcal{l}_{text}(C)+\mathcal{l}_{audio}(C)) \end{aligned}$ (4)

where $\begin{aligned} \mathcal{l}_{k}=\frac{1}{N}\begin{aligned} \sum_{i=0}^{N} log \end{aligned} (diag(softmax\left( C \right))) \end{aligned}$ along text ($\mathcal{l}_{text}$) and audio ($\mathcal{l}_{audio}$) axis respectively. We used this symmetric cross-entropy loss ($\mathcal{L}$) over the similarity matrix to jointly train the audio and text encoders along with their linear projections.


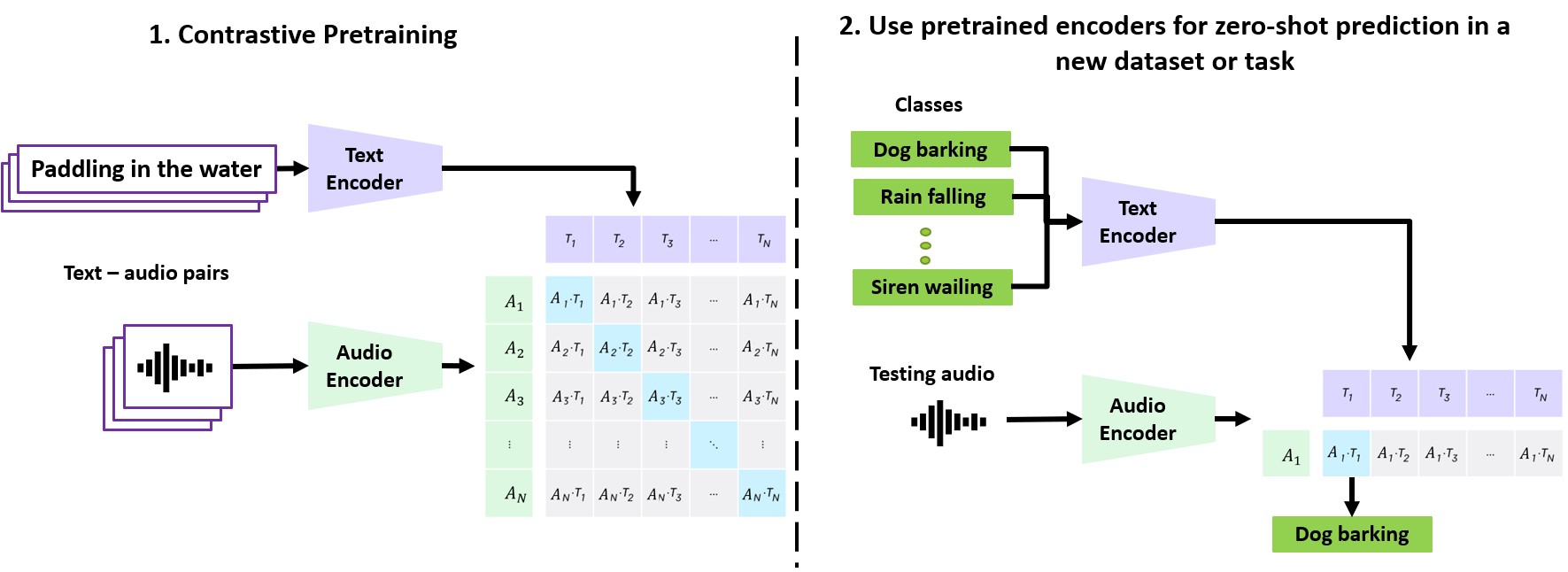


Supplementary Figure 1: Technically, CLAP uses two encoders (an audio feature encoder and a language feature encoder) and a contrastive learning objective, bringing audio and text descriptions into a joint multimodal space. On the pretraining stage, input audio and corresponding text descriptions are passed to an audio encoder and a text encoder respectively to generate audio and text feature embeddings. Both embeddings are connected in a joint multi-modal space with a linear projection. The multi-modal space is learned with the (dis)similarity of the audio and text feature embeddings in each training batch using a contrastive learning loss. The model is based on our previous work [2], in which we evaluated CLAP on 16 downstream tasks across 7 domains, such as classification of sound events, scenes, music, and speech.

### Zero-Shot Transfer

For the inference stage, the pretrained encoders with their projection layers are used to compute audio and text embeddings of the audio recordings and the class labels of a target dataset. Zero-Shot Classification is performed by computing cosine similarity between each audio file in the testing split and all the class labels. No training on the target dataset is needed.

For Zero-Shot classification, we used CLAP’s ability to determine the similarity between audio and text. Let’s consider a target dataset with *C* class labels and *N* test audios. First, we compute audio embeddings and text embeddings for *N* audios and *C* classes using the pretrained encoders and their projection layers. Second, because both the embeddings are in a common space, we compute the cosine similarity between each testing audio and all the class labels. Each audio will have as many logits as class labels. Third, logits are turned into a probability distribution by applying softmax for binary or multi-class and sigmoid for multi-label classification. Note that there is no additional training stage for any set of *N* audios and *C* classes.

## Details of pretraining CLAP and Zero-Shot Transfer

**Audio pre-processing.** We used log Mel spectrogram representations of audio segments with a sampling rate of 44.1 KHz, hop size of 320 frames, window size 1024 frames, and 64 Mel bins in the range of 50-8000 Hz. During training, each audio clip is randomly truncated to a continuous segment of 7 secs, or padded if shorter. The captions were not altered. The batches with audio and text pairs are randomly sampled at training.

**Audio and Text Encoders.** We chose CNN14 [3] and HTS-AT [4] model as the audio encoder due to its SoTA performance. CNN14 has 80M and HTS-AT has 30M parameters, an embedding size of 2048, and was pretrained with 2M audio clips from AudioSet. The text encoder is the HuggingFace [5] implementation of BERT base uncased for CNN14 and CLIP text encoder [6] for HTS-AT. Both audio encoder and text encoder are fine-tuned during training. We limited the max text sequence length to 100 tokens for computational efficiency. The [CLS] token from the final layer of BERT is used as the text embedding with a size of 768. Both, the audio and text embeddings are projected into a multi-modal space with two learnable projection matrices resulting in an output dimension of 1024. The temperature parameter *τ* is learnable and initialized to 0.007. To prevent training instability, the logits scaled by *τ* are clipped to a maximum value of 100.

**Pretraining.** We trained by unfreezing both encoders for 40 epochs. We used the CLAP model from the epoch that yielded the best performance in FSD50k. We use Adam Optimizer with an initial learning rate 10^´3^ and reduce the learning rate on plateau by 10^´1^ with a patience of 10. The models are implemented with PyTorch’s Distributed Data-Parallel and used 16GB V100 GPUs with scaling from 8 to 24 GPUs. Batch size was 128.

**Zero-Shot Transfer** During inference/testing, the CLAP model has never seen in pretraining the audio from the benchmark datasets. Instead of using the class label, we constructed a natural language prompting. The text prompts used for each benchmark datasets are reported in Table 1 in the main text.

## Evaluation metric

Since our task is mostly binary classifications, we use a widely applied evaluation metric, Average Precision (AP) as our performance evaluation metric [7, 8]. And we only report the AP of our target class (i.e., positive class) in the project. We calculate AP as following calculation:

$\begin{aligned} AP=\sum_{n} (R_{n}-R_{n-1})P_{n} \end{aligned}$(5)

where *P_n_* and *R_n_* are the precision and recall at the *n_th_* threshold [9].

## Details of pretraining datasets

To pretrain the three versions of CLAP model, we extracted audio-text pairs from different datasets and web archives:

- CLAP-PANN (128K): FSD50k [10], ClothoV2 [11], AudioCaps [12], and MACS [13].
- CLAP-HTS-AT (450K): 128k plus SoundDescs [14], BigSoundBank [15], SoundBible [15], FMA [16], NSynth [17], findsound.com.
- CLAP-HTS-AT (2.1M): 450k plus CMUMOSI [18], MELD [19], IEMOCAP [20], MOSEI [18], MSPPodcast [21], CochlScene [22], AudioSet (Filtered) [23], Kinetics700 [24], Freesound [25], ProSoundEffects [26].

The datasets come from different domains containing environmental sounds, speech emotions, actions, and music. Although these datasets were not specifically annotated for bioacoustics research, they encompass a wide range of animal sounds. This diversity enables the Zero-Shot Transfer of CLAP to bioacoustics tasks.

In Supplementary Table 1, we exemplify the variability in the complexity of captions across the datasets. The captions in Clotho contain multiple sound events like *“crow crows and a person imitating the crow”* and the same in MACS *“a crow screaming with kids yelling in the background along with another bird”*. SoundDescs has larger captions with multiple events in longer-duration audio recordings, the WavText5K [15] caption focuses on *“crow crying”* and provides a description of where and when (*“the middle of the night”*) the event is happening.

| Source | Description |
| --- | --- |
| AudioCaps | *Screeching and light banging with a distant crow calling.* |
| Clotho | *A crow crows loudly as a person is heard imitating the sound.* |
| MACS | *a crow is screaming in the background kids are yelling then another bird is screaming.* |
| SoundDescs | *Birds - Birds, Madumbalai National Park, early*  *morning with close-up partridge calls, warblers, crow-pheasants and house crow* |
| WavText5K | *A single crow crying in the middle of the night* |

Supplementary Table 1: Variability of captions across 5 datasets.

## Details of the 9 bioacoustics benchmark datasets

For all the audio segments, we first resampled the samples to 44.1kHz to match the training data of CLIP. We then computed log Mel spectrogram representations using the same parameters for the training data: hop size of 320 frames, window size 1024 frames, and 64 Mel bins in the range of 50-8000 Hz. For training the supervised baseline models, we also follow the BEANS protocol to randomly augment the spectrograms for better supervised classification performance.

The technical details of the datasets are in Supplementary Table 2. Tropical Gunshots dataset does not have a test set. The supervised model was validated and tested on the same validation set. CLAP was also tested on the validation set. Warblr, Freefield, and Tropical Gunshots are pre-segmented datasets. Therefore, they do not have step sizes. The *# Categories* columns in Supplementary Table 2 represent the number of independent categories (e.g., species of birds) besides noise.

Supplementary Table 2: Technical details of the bioacoustics datasets in this project.

| Dataset | Subject | # Categories | Train/Val/Test # | | Default sampling rate (Hz) | Window size | Step size |
| --- | --- | --- | --- | --- | --- | --- | --- |
| Jackdaw [27] | Birds | 1 | 413/118/59 | 16000 | | 2 sec. | 1 sec. |
| Enabirds [28] | Birds | 34 | 13629/4543/4543 | 32000 | | 2 sec. | 1 sec. |
| Freefield [29] | Birds | - | 5383/1153/1154 | 44100 | | 10 sec. | - |
| Warblr [30] | Birds | - | 5600/1200/1200 | 44100 | | 10 sec. | - |
| Rfcx [31] | Birds & Frogs | 24 | 2334/764/711 | 48000 | | 10 sec. | 5 sec. |
| Hiceas [8] | Minke Whales | 1 | 4457/1458/1458 | 22050 (down-sampled from 500000 by BEANS) | | 10 sec. | 5 sec. |
| Meerkat [27] | Meerkats | 2 | 2773/944/741 | 16000 | | 2 sec. | 1 sec. |
| Tropical Gunshots [32] | Gunshots | 1 | 28790/7190/- | 800 | | 4 sec. | - |

# References

1. R. Bommasani, D. A. Hudson, E. Adeli, R. Altman, S. Arora, S. von Arx, M. S. Bernstein, J. Bohg, A. Bosselut, E. Brunskill *et al.*, “On the opportunities and risks of foundation models,” *arXiv preprint arXiv:2108.07258*, 2021.
2. B. Elizalde, S. Deshmukh, M. A. Ismail, and H. Wang, “Clap: Learning audio concepts from natural language supervision,” *arXiv preprint arXiv:2206.04769*, 2022.
3. Q. Kong, Y. Cao, T. Iqbal, Y. Wang, W. Wang, and M. D. Plumbley, “Panns: Large-scale pretrained audio neural networks for audio pattern recognition,” *IEEE/ACM Transactions on Audio, Speech, and Language Processing*, vol. 28, pp. 2880–2894, 2020.
4. K. Chen, X. Du, B. Zhu, Z. Ma, T. Berg-Kirkpatrick, and S. Dubnov, “Hts-at: A hierarchical token-semantic audio transformer for sound classification and detection,” in *ICASSP 2022-2022 IEEE International Conference on Acoustics, Speech and Signal Processing (ICASSP)*. IEEE, 2022, pp. 646–650.
5. T. Wolf, L. Debut, V. Sanh, J. Chaumond, C. Delangue, A. Moi, P. Cistac, T. Rault, R. Louf, M. Funtowicz *et al.*, “Huggingface’s transformers: State-of-the-art natural language processing,” *arXiv preprint arXiv:1910.03771*, 2019.
6. A. Radford, J. W. Kim, C. Hallacy, A. Ramesh, G. Goh, S. Agarwal, G. Sastry, A. Askell, P. Mishkin, J. Clark *et al.*, “Learning transferable visual models from natural language supervision,” in *International conference on machine learning*. PMLR, 2021, pp. 8748–8763.
7. Z. Dong, K. Xu, Y. Yang, H. Bao, W. Xu, and R. W. Lau, “Location-aware single image reflection removal,” in *Proceedings of the IEEE/CVF International Conference on Computer Vision*, 2021, pp. 5017–5026.
8. M. Hagiwara, B. Hoffman, J.-Y. Liu, M. Cusimano, F. Effenberger, and K. Zacarian, “Beans: The benchmark of animal sounds,” in *ICASSP 2023-2023 IEEE International Conference on Acoustics, Speech and Signal Processing (ICASSP)*. IEEE, 2023, pp. 1–5.
9. F. Pedregosa, G. Varoquaux, A. Gramfort, V. Michel, B. Thirion, O. Grisel, M. Blondel, P. Prettenhofer, R. Weiss, V. Dubourg, J. Vanderplas, A. Passos, D. Cournapeau, M. Brucher, M. Perrot, and E. Duchesnay, “Scikit-learn: Machine learning in Python,” *Journal of Machine Learning Research*, vol. 12, pp. 2825–2830, 2011.
10. E. Fonseca, X. Favory, J. Pons, F. Font, and X. Serra, “Fsd50k: An open dataset of human-labeled sound events,” *IEEE/ACM Transactions on Audio, Speech, and Language Processing*, 2022.
11. K. Drossos, S. Lipping, and T. Virtanen, “Clotho: an audio captioning dataset,” in *IEEE International Conference on Acoustics, Speech and Signal Processing (ICASSP)*, 2020.
12. C. D. Kim, B. Kim, H. Lee, and G. Kim, “AudioCaps: Generating Captions for Audios in The Wild,” in *NAACL-HLT*, 2019.
13. I. Mart´ın-Morato´ and A. Mesaros, “What is the ground truth? reliability of multi-annotator data for audio tagging,” in *2021 29th European Signal Processing Conference (EUSIPCO)*, 2021.
14. A. S. Koepke, A.-M. Oncescu, J. Henriques, Z. Akata, and S. Albanie, “Audio retrieval with natural language queries: A benchmark study,” *IEEE Transactions on Multimedia*, 2022.
15. S. Deshmukh, B. Elizalde, and H. Wang, “Audio retrieval with wavtext5k and clap training,” *arXiv preprint arXiv:2209.14275*, 2022.
16. M. Defferrard, K. Benzi, P. Vandergheynst, and X. Bresson, “Fma: A dataset for music analysis,” *arXiv preprint arXiv:1612.01840*, 2016.
17. J. Engel, C. Resnick, A. Roberts, S. Dieleman, D. Eck, K. Simonyan, and M. Norouzi, “Neural audio synthesis of musical notes with wavenet autoencoders,” 2017.
18. A. B. Zadeh, P. P. Liang, S. Poria, E. Cambria, and L.-P. Morency, “Multimodal language analysis in the wild: Cmu-mosei dataset and interpretable dynamic fusion graph,” in *Proceedings of the 56th Annual Meeting of the Association for Computational Linguistics (Volume 1: Long Papers)*, 2018, pp. 2236–2246.
19. S. Poria, D. Hazarika, N. Majumder, G. Naik, E. Cambria, and R. Mihalcea, “Meld: A multimodal multi-party dataset for emotion recognition in conversations,” *arXiv preprint arXiv:1810.02508*, 2018.
20. C. Busso, M. Bulut, C.-C. Lee, A. Kazemzadeh, E. Mower, S. Kim, J. N. Chang, S. Lee, and S. S. Narayanan, “Iemocap: Interactive emotional dyadic motion capture database,” *Language resources and evaluation*, vol. 42, no. 4, pp. 335–359, 2008.
21. R. Lotfian and C. Busso, “Building naturalistic emotionally balanced speech corpus by retrieving emotional speech from existing podcast recordings,” *IEEE Transactions on Affective Computing*, vol. 10, no. 4, pp. 471–483, 2017.
22. I.-Y. Jeong and J. Park, “Cochlscene: Acquisition of acoustic scene data using crowdsourcing,” in

*2022 Asia-Pacific Signal and Information Processing Association Annual Summit and Conference (APSIPA ASC)*. IEEE, 2022, pp. 17–21.

1. J. F. Gemmeke, D. P. W. Ellis, D. Freedman, A. Jansen, W. Lawrence, R. C. Moore, M. Plakal, and M. Ritter, “Audio set: An ontology and human-labeled dataset for audio events,” in *2017 IEEE International Conference on Acoustics, Speech and Signal Processing (ICASSP)*, 2017, pp. 776–780.
2. W. Kay, J. Carreira, K. Simonyan, B. Zhang, C. Hillier, S. Vijayanarasimhan, F. Viola, T. Green, T. Back, P. Natsev *et al.*, “The kinetics human action video dataset,” *arXiv preprint arXiv:1705.06950*, 2017.
3. V. Akkermans, F. Font Corbera, J. Funollet, B. De Jong, G. Roma Trepat, S. Togias, and X. Serra, “Freesound 2: An improved platform for sharing audio clips,” in *Klapuri A, Leider*

*C, editors. ISMIR 2011: Proceedings of the 12th International Society for Music Information Retrieval Conference; 2011 October 24-28; Miami, Florida (USA). Miami: University of Miami; 2011.* International Society for Music Information Retrieval (ISMIR), 2011.

1. M. Hanish, “Pro sound effects’ hybrid sound effects library,” *TV Technology*, 2015.
2. V. Morfi, I. Nolasco, V. Lostanlen, S. Singh, A. Strandburg-Peshkin, L. F. Gill, H. Pamula, D. Benvent, and D. Stowell, “Few-shot bioacoustics event detection: A new task at the dcase 2021 challenge.” in *DCASE*, 2021, pp. 145–149.
3. L. M. Chronister, T. A. Rhinehart, A. Place, and J. Kitzes, “An annotated set of audio recordings of eastern north american birds containing frequency, time, and species information,” 2021.
4. D. Stowell and M. D. Plumbley, “An open dataset for research on audio field recording archives: freefield1010,” *arXiv preprint arXiv:1309.5275*, 2013.
5. D. Stowell, M. Wood, Y. Stylianou, and H. Glotin, “Bird detection in audio: a survey and a challenge,” in *2016 IEEE 26th International Workshop on Machine Learning for Signal Processing (MLSP)*. IEEE, 2016, pp. 1–6.
6. J. LeBien, M. Zhong, M. Campos-Cerqueira, J. P. Velev, R. Dodhia, J. L. Ferres, and T. M. Aide,

“A pipeline for identification of bird and frog species in tropical soundscape recordings using a convolutional neural network,” *Ecological Informatics*, vol. 59, p. 101113, 2020.

1. L. K. Katsis, A. P. Hill, E. Pina-Covarrubias, P. Prince, A. Rogers, C. P. Doncaster, and J. L. Snaddon, “Automated detection of gunshots in tropical forests using convolutional neural networks,” *Ecological Indicators*, vol. 141, p. 109128, 2022.
